# Supplementary material for: The genome sequence of the solitary wasp, Cerceris ruficornis (Fabricius, 1793)
Source: Wellcome Open Res. 2025 May 27;10:276. [Version 1] doi: 10.12688/wellcomeopenres.24270.1 (PMC12411840; doi:10.12688/wellcomeopenres.24270.1)
Supplement: Peer review history

# Reviewer report: Reviewer response for version 1

**DOI:** 10.21956/wellcomeopenres.26767.r126007

**Published:** 2025-11-05

**Authors:** Kashif Zahoor

**Affiliations:** 1 Government College University Faisalabad, Faisalabad, Punjab, Pakistan

Its really huge work, and well presented.

 However, I've few comments: 

 "Most of the assembly sequence (65.35%) was assigned to 14 chromosomal-level scaffolds. These chromosome-level scaffolds, confirmed by Hi-C data, are named according to size"

 Must cite some references? 

 How assembly sequence was assigned to 14 chromosomal-level scaffolds?? 

 How genetic data was assigned to respective chromosomes?

 How chromosomes were annotated with numbering? How chromosome mapping can be verified?

 its only *in silico *which is possible at this stage?....  which protocol was followed for assigning/annotation of chromosomes? Its Rhie et al., 2021??

 Mitochondrial gene COI was used only or other marker like COII, ND4, etc  was used? If Mitochondria complete genome is 18.07kb? 

 Which "Tissue" was used? 

 GC% should be verified? or only If BUSCO scores are sufficient when compared with other Hymenoptera?  

 I recommend this manuscript for indexing after incorporating to my comments

Are sufficient details of methods and materials provided to allow replication by others?

Yes

Is the rationale for creating the dataset(s) clearly described?

Yes

Are the datasets clearly presented in a useable and accessible format?

Yes

Are the protocols appropriate and is the work technically sound?

Yes

Reviewer Expertise:

Genetics, Molecular Biology, Entomology

I confirm that I have read this submission and believe that I have an appropriate level of expertise to confirm that it is of an acceptable scientific standard.

# Reviewer report: Reviewer response for version 1

**DOI:** 10.21956/wellcomeopenres.26767.r130386

**Published:** 2025-09-08

**Authors:** Sara E Miller

**Affiliations:** 1 Department of Biology, University of Missouri St. Louis, Saint Louis, MO, USA; 2 Cornell University, Missouri, USA

The authors present a new high quality nuclear and mitochondrial genome assembly for the solitary wasp, *Cerceris ruficornis* (Hymenoptera: Crabronidae). This is the first genome assembly for this species and adds to the available genomes for this large family of wasps.

 Genome sequencing and assembly were completed using standard protocols of the Darwing Tree of Life Consortium.  The genome was constructed using single female using Pacific Biosciences HiFi circular consensus DNA libraries, sequenced on the Biosciences SEQUEL II, and scaffolded with Hi-C data. Raw data and assembly methods are available. The genome assembly is contiguous and high quality with a scaffold N50 of 20.57 Mb and 96.5% coverage of BUSCO genes. An annotation is provided with this genome.

 The final assembly is on the larger side for Hymenopteran genomes, at 566.08 Mb, and only 65.35% of the genome was able to be assigned to chromosomal-level scaffolds. Presumably the increase of genome size and difficulty resolving portions of this genome into chromosomal-level scaffolds is the result of expansion of repetitive sequences. However, the number and distribution of repetitive elements is not directly addressed by the authors and feels like a missed opportunity here.

 Overall, this is a standard genome report and can be indexed as is.

Are sufficient details of methods and materials provided to allow replication by others?

Yes

Is the rationale for creating the dataset(s) clearly described?

Yes

Are the datasets clearly presented in a useable and accessible format?

Yes

Are the protocols appropriate and is the work technically sound?

Yes

Reviewer Expertise:

Hymenoptera genetics and genomics, evolutionary ecology

I confirm that I have read this submission and believe that I have an appropriate level of expertise to confirm that it is of an acceptable scientific standard.

# Reviewer report: Reviewer response for version 1

**DOI:** 10.21956/wellcomeopenres.26767.r126582

**Published:** 2025-09-05

**Authors:** Alexandra Cerqueira De Araujo

**Affiliations:** 1 University College London, London, UK

The authors present here a chromosome level *de novo* assembly of the genome of *Cerceris ruficornis *(Crabronidae, Hymenoptera), first assembly available for this species. This assembly is composed of 14 chromosomes and one mitochondrial chromosome, with an assembly total length of 566.08Gb and a N50 of 20.57Mb. This assembly achieves a EBP reference standard of 6.7.Q65 and contains most of the orthologous genes found in Hymenoptera (i.e. 96.5% of the complete BUSCO sequences). Altogether, these values reflect an assembly quality that is above the minimum reference standard for genome assemblies.

 Although I deem this paper adequate for indexing, however I would like to raise a few comments that should be addressed before indexing:

1. In the “Sequencing data” paragraph and Table 1, the read count for ERR12102416 (i.e. 5.45e+08) doesn’t match the read count reported on NCBI (2.7e+08 reads reported by NCBI).
2. The authors have assembled the genome of *C. ruficornis* to chromosome level. They managed to obtained 14 chromosomes in total, plus extra sequences (i.e. 559 scaffolds). Is there any reference supporting this number of chromosomes for this species?
3. The assembly span for the genome of *C. ruficornis* is bigger than the span of most hymenopteran genomes (mean span = 338Gb, median span = 287Gb). There is also only 65% of the total assembly contained in the 14 chromosomes. How many genes, especially BUSCO genes, are present in these 14 chromosomes? I found in total 11093 sequences with the attribute “gene” in the gff provided in https://beta.ensembl.org/species/060ab491-5335-4f09-95ba-32ffa082ec0b, 11073 of them were found in the 14 chromosomes. I believe that the author should emphasis this results as it shows that almost the entirety of coding regions is contained in the assembled 14 chromosomes. The genome size (in GB) could be smaller than the span mentioned in the manuscript. Of the remaining scaffolds, how many of them align to other scaffold in the assembly? i.e. how many of them are repeated?
4. I see some discrepancies in the “Genome annotation report” section:
  1. The average number of coding transcript per gene is 1.50 in the manuscript, but the number found in https://beta.ensembl.org/species/060ab491-5335-4f09-95ba-32ffa082ec0b is 1.57.
  2. The average transcript length is 11,558.03 bp in the manuscript, but this value cannot be found in https://beta.ensembl.org/species/060ab491-5335-4f09-95ba-32ffa082ec0b. Using AGAT v1.4.1 (agat_sp_statistics.pl), I found: mean mRNA length = 12404 bp, mean gene length = 12138 bp, mean number of exons per mRNA  = 6.6 (but 6.2 in the manuscript).
5. The methods and protocols used to build and analyse the assembly are I believe appropriate. However, in its current state, it is not possible to build the exact same assembly built by the authors, as parameter information is missing. I do not see any link to code repository to generate this assembly and all data associated to it (did I miss it?). If the authors would prefer to not upload the code to an online repository, it is important that they mention the parameters used for all programs and tools mentioned in the manuscript (I believe they could be added to Table 4). If I did miss the link, please, make it more visible. I did see this link https://zenodo.org/records/10047654 , which is associated to this sentence “Flat files and maps used in curation were generated via the TreeVal pipeline ( Pointon *et al.*, 2023).” in the “Assembly curation” section, but it is not clear whether this pipeline has been used only for this section or not.

Are sufficient details of methods and materials provided to allow replication by others?

Partly

Is the rationale for creating the dataset(s) clearly described?

Yes

Are the datasets clearly presented in a useable and accessible format?

Yes

Are the protocols appropriate and is the work technically sound?

Partly

Reviewer Expertise:

Comparative genomics

I confirm that I have read this submission and believe that I have an appropriate level of expertise to confirm that it is of an acceptable scientific standard.

# Reviewer report: Reviewer response for version 1

**DOI:** 10.21956/wellcomeopenres.26767.r130387

**Published:** 2025-09-04

**Authors:** Sarah Inwood

**Affiliations:** 1 University of Otago, Dunedin, New Zealand

The authors have used state-of-the-art sequencing and assembly methods to generate a genome for the solitary wasp *Cerceris ruficornis.* The genome was generated from a single individual of presumably unknown sex (this is not stated) using both HiFi and Hi-C data with BUSCO scores suggestive of a high-quality assembly. The authors also produced a mitochondrial genome assembly.

**Minor comments:**

 Only 65.35% of the genome was scaffolded into chromosomal pseudomolecules and given the stated benchmark for this is above 90%, the lack of discussion of this result was surprising. Figure 3 shows that all of the scaffolded chromosomal pseudomolecules had hits to Arthropoda while none of the smaller unscaffolded contigs do, with these appearing to be in a couple of fairly distinct clusters. Given they could potentially be contaminants not detected by the ASCC pipeline, it would be of interest to know whether any of the unscaffolded contigs contained BUSCO hits or any other data to suggest they definitely belong in the genome assembly of *Cerceris ruficornis, *or whether the only data to suggest they belong in the assembly was them not being filtered out by the ASCC pipeline.

 The Hi-C contact map in Figure 5 also looks a bit odd, with strong contacts between the ends of most chromosomal pseudomolecules. This result would also benefit from acknowledgement and brief discussion, whether it can be linked to something else e.g. is it repeat sequence, a potential artifact, or does it have no explanation? Chromosome 4 also seems to have interactions with a number of the unscaffolded contigs; this too could benefit from discussion - are these thought to be the result of incomplete scaffolding, or is there evidence to support them not being scaffolded?

Are sufficient details of methods and materials provided to allow replication by others?

Yes

Is the rationale for creating the dataset(s) clearly described?

Yes

Are the datasets clearly presented in a useable and accessible format?

Partly

Are the protocols appropriate and is the work technically sound?

Yes

Reviewer Expertise:

Genomics, host-parasite interactions, parasitoid wasps, insects, viruses, biocontrol

I confirm that I have read this submission and believe that I have an appropriate level of expertise to confirm that it is of an acceptable scientific standard, however I have significant reservations, as outlined above.

# Reviewer report: Reviewer response for version 1

**DOI:** 10.21956/wellcomeopenres.26767.r130391

**Published:** 2025-08-30

**Authors:** Ariel Chipman

**Affiliations:** 1 Hebrew University of Jerusalem School of Pharmacy, Jerusalem, Jerusalem District, Israel

Sivell et al. report on the genome sequence of the solitary wasp *Cerceris ruficornis*, a species known from much of Northern Europe. The specimen they sequenced was collected in Norfolk, England.

 The sequencing and assembly were done according to the standard protocols and metrics of the Darwin Tree of Life Consortium. The quality of the assembly is good but falls short of the benchmark in some of the metrics, specifically in the percentage of the sequence successfully assembled into chromosomes. BUSCO and completeness scores are high.

 The report is fairly standard. It is written clearly and according to the guidelines for such a report. I see no reason not to accept the report as it is.

Are sufficient details of methods and materials provided to allow replication by others?

Yes

Is the rationale for creating the dataset(s) clearly described?

Yes

Are the datasets clearly presented in a useable and accessible format?

Yes

Are the protocols appropriate and is the work technically sound?

Yes

Reviewer Expertise:

Arthropods evolution, evo-devo comparative genomics.

I confirm that I have read this submission and believe that I have an appropriate level of expertise to confirm that it is of an acceptable scientific standard.

# Reviewer report: Reviewer response for version 1

**DOI:** 10.21956/wellcomeopenres.26767.r125320

**Published:** 2025-07-12

**Authors:** Guanliang Meng

**Affiliations:** 1 Leibniz Institute, Berlin, Germany

The authors use PacBio HiFi, Hi-C data to assemble the genome of *Cerceris ruficornis*, anchoring the scaffolds into 14 chromosomes, providing haplotype-level assemblies. Gene completeness analysis such as BUSCO scores suggest that the genome is high quality. My major concerns include:

 (1) The differences between estimated genome size (454.59 Mb) and the assembled size (566.08 Mb);

 (2) only 65.35% of the assembly was assigned to the 14 chromosomes. What are the true number of chromosomes for the species? What are the un-assigned scaffolds? Do they belong to contaminants (perhaps the ASCC pipeline was not able to identify these contaminants)? Especially that there are several clouds of dots in Figure 3;

 (3) The intensive links between the ends of chromosomes (Figure 5). Are these due to assembly error, or due to wrong placements of the scaffolds, or due to repeats such as telomeres?

Are sufficient details of methods and materials provided to allow replication by others?

Yes

Is the rationale for creating the dataset(s) clearly described?

Yes

Are the datasets clearly presented in a useable and accessible format?

Yes

Are the protocols appropriate and is the work technically sound?

Yes

Reviewer Expertise:

Genomics, Transcriptomics, Bioinformatics, Evolutionary Biology

I confirm that I have read this submission and believe that I have an appropriate level of expertise to confirm that it is of an acceptable scientific standard, however I have significant reservations, as outlined above.
